# Organoids in recapitulating tumorigenesis driven by risk factors: Current trends and future perspectives

**DOI:** 10.7150/ijbs.70406

**Published:** 2022-03-28

**Authors:** Jie Zhang, Lei Wang, Qianqian Song, Mingbing Xiao, Jie Gao, Xiaolei Cao, Wenjie Zheng

**Affiliations:** 1 Research Center of Clinical Medicine, Affiliated Hospital of Nantong University, 20 Xisi Road, 226001 Nantong, Jiangsu, China.; 2 Department of Radiology, Wake Forest School of Medicine, One Medical Center Boulevard, Winston-Salem, 27157 NC, USA.; 3 School of Medicine, Nantong University, 19 Qixiu Road, 226001 Nantong, Jiangsu, China.

**Keywords:** Organoid, Carcinogenesis, Risk factors, Robust model, Prospect outlook

## Abstract

Environmental and exogenous/ endogenous factors, in a setting of individual genetic predisposition, contribute to the cancer development. Over the years, epidemical evidence increasingly highlights the correlations of multiple cancer incentives and genetic alterations with cancer incidence. Unraveling the pivotal carcinogenesis events prompted by particular risk factors remarkably advances early surveillance and oncogenesis intervening. Traditional cell-based models and animal-based models are unrealistic and unreliable for translational study, respectively ascribing to the limited tumor heterogeneity and species-related variation. Organoid emerged as a fidelity model that well preserves the properties of its origin. With inherent quality of holistic perspective, organoid is therefore ideally suited for delineating the carcinogenesis under risk exposure, in favor of understanding pathogen-host interactions and alleviating cancer initiation. In this review, we have summarized the organoid model-based evidence that identified or validated carcinogenic risks, mainly including diet, aging, microbial infection, and chemical exposure. In addition, we envisioned the exciting prospect of organoid model in screening promising treatment and/or prevention during tumorigenesis. As a robust 3D *in vitro* system, organoid has been widespread applied in basial and clinical cancer research, which may elucidate crucial mechanisms of oncogenesis and develop novel targeting strategies.

## Introduction

Cancer is a common leading cause of death globally. It is expected to 28.4 million new cancer cases (except basal cell carcinoma) worldwide by 2040, which leads to a 47% elevation from the cases in 2020 1. As the latest statistics, the burden of neoplastic disease is increasing in a majority of nations, with 64% to 95% in transitioning versus 32% to 56% in transitioned countries. This reflects both aging and growth of the population, as well as the increasing risk factors for cancers mainly associated with socioeconomic development, which include dietary bias, smoking, ultraviolet light, environmental chemicals, and pathogen exposure. Thereby, nascent strategies are urgent for cancer prevention. Identifying tumorigenesis- promoting factors and revealing the potential mechanisms might hinder the course of carcinogenesis. Besides, clinical observations have indicated that cancer treatment at early stages is more effective as early intervention may successfully disrupt carcinogenic pathways.

In past decades, distinct models were developed for studying tumor initiation, typically cell-based models *in vitro* and animal- based models* in vivo*. Though animal models better mimicry of carcinogenesis, it is time-costing, low-repeated, more importantly limited with species-related variation. Constructing a low-costing and high-repeated model that maximumly mimic the original tissue is expected to identify tumor-initialing factors and investigate potential mechanisms. For this, organoids are mini-organs reconstituted and embedded in an extra-cellular matrix. The initial organotypic models were reported to generate cortical and intestinal tissues in 2008 and 2009, respectively 2, 3. In 2009, intestinal crypt villus organoid was generated from Lgr5(+) stem cells located at the bottom of intestinal crypts which were isolated from mouse intestinal tissue 3. The Lgr5+ cells were embedded in Matrigel (a gelatinous protein matrix that provides the structural architecture to support 3D growth) and overlaid with a specialized culture medium bearing several growth factors. The resulting organoid was shaped as a central lumen surrounded by a monolayer of epithelial cells and protrusions of the stem cells niche consisting of stem cells and differentiated Paneth cells. Thereafter, this novel technology was also adapted to generate organoid from human small intestine 4. To date, efficient and robust protocols have been established for tissues covering colon 5, liver 6, pancreas 7, brain 8, breast 9, prostate 10, and ovaries 11. Organoids have been developed to model multiple diseases, such as cystic fibrosis and Zika virus infection 12, neurodevelopmental disorders 13-16, and chronic liver diseases 17. In addition, they can be stored as “living biobanks” 18 and employed in drug testing 19, 20. The advent of patient-derived organoids (PDOs), characterized by high-fidelity of the original tumors, represents a prospective opportunity for basic and translational studies. Organoid technology circumvents not only historic drawbacks of cell lines of lacking TME and tissue heterogeneity, but also weak scalability of organismal models, such as mice, rats, zebrafish, and Drosophila 21.

Emerging evidence confirmed organoid as an ideal 3D *in vitro* system for monitoring cancer oncogenesis. Starting from primary sources of normal cells, followed by a few passages, the organoid's oncogenic genes accumulate and ultimately drive malignant transformation. This progress resembles genuine cancer oncogenesis 22. Importantly, organoid models from stem cells are capable of tracking and manipulating genomes, transcriptomes, and epigenomes, fueling the study of specific alterations and milieu factors during tumorigenesis. This burgeoning culture technology represents a high-fidelity tool to fabricate the process of cancer formation and progression. Organoids co-cultured with stromal components allow for investigating into the complexity of inflammation-to-tumor microenvironment (TME). Furthermore, integration of different environmental components to organoids effectuates the determination of risk factors for tumor formation or protective agents against neoplasm initiation.

## Organoids focusing on carcinogenesis

**Figure 1:**
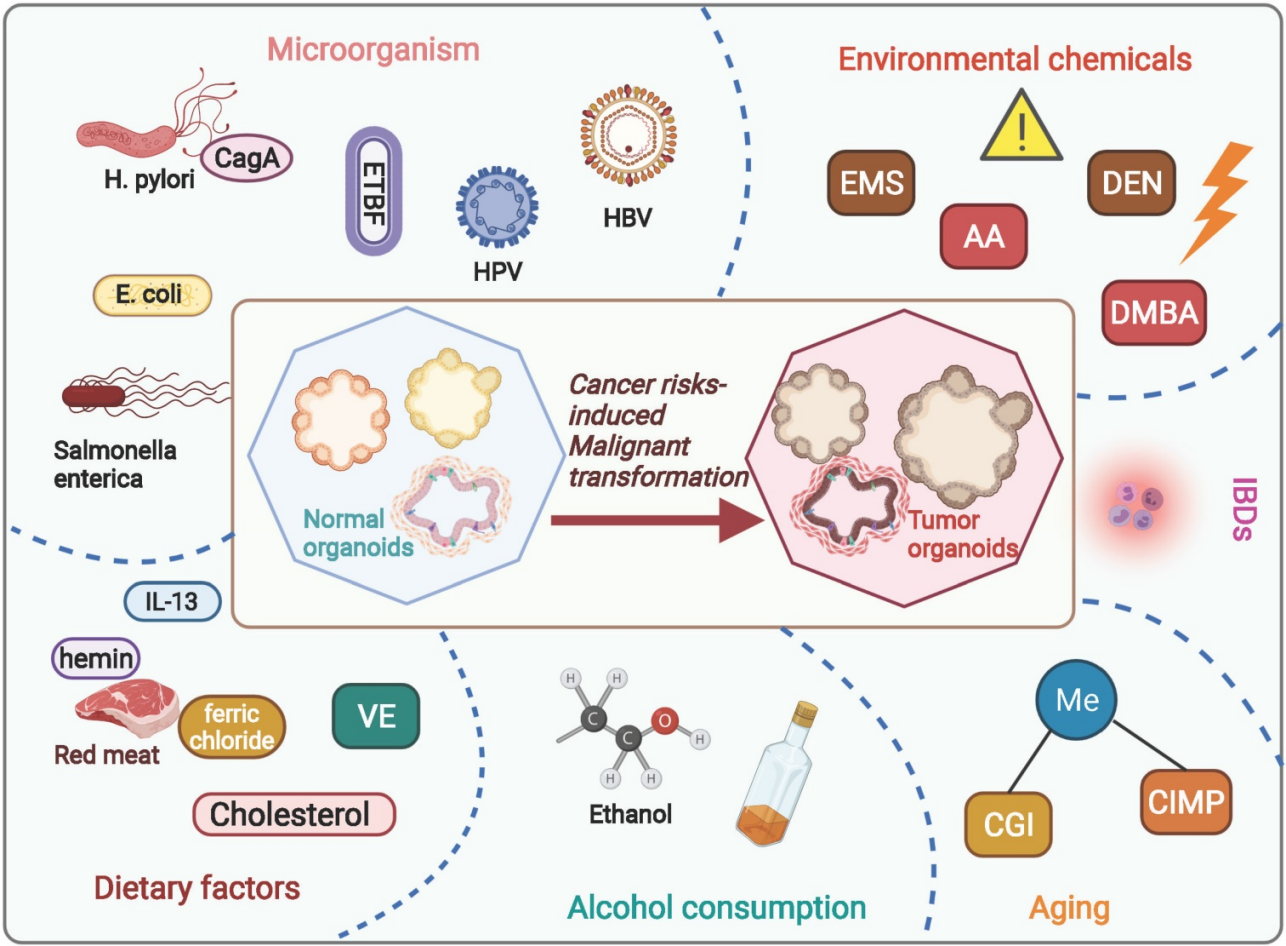
**Current studies regarding cancer risks-induced tumorigenesis based on organoid model.** By using organoid system, numerous studies have focused on investigating risk factors induced carcinogenesis, including dietary risk factors, alcohol consumption, aging, environmental chemicals, microorganisms' infections, and inflammatory bowel diseases. **AA**, acrylamide; **EMS**, ethyl methanesulfonate; **DEN**, diethylnitrosamine; **DMBA**, 7,12-dimethylbenz[a]anthracene;** CGI**, CpG-island; **CIMP**, CpG island methylator phenotype; ***E. coli***, *Escherichia coli*; ***H. pylori***, *Helicobacter pylori*; **ETBF**, Enterotoxigenic Bacteroides fragilis; **CagA**, cytotoxin-associated gene A; **HPV**, human papillomavirus; **VE**, Vitamin E; **IBDs**, Inflammatory bowel diseases.

Carcinogenesis is a complex process initiate from stem cells experiencing a collection of endogenetic and epigenetic variations influenced by external environmental factors over the years. Through this course, neoplasm formation is endowed with a high degree of heterogeneity in tumor cells as well as in the milieu, bringing up cancer treatment challenging. A number of risk factors have been proved responsible for the development of malignant neoplasia among humans, leading with smoking, excessive alcohol consumption, diet, and reproductive behavior 23. Elucidating the molecular characteristics and risk factors involved in neoplasm development will enhance diagnostic information to inform therapeutic actions. Organoid, well-preserving the heterogenous property of protocols, provides a superior model for delineating the carcinogenesis under natural or anthropogenic exposures **(Figure 1 and Table 1)**.

### Dietary risk factors

#### High fat diet (HFD)

Obesity has been recognized as a major risk factor for a wide range of human cancers, with rising rates, including colorectal cancer (CRC). However, it is obscure that how pro-obesity diet influences the stem cells and progenitors to alter their intrinsic potential for tumor initiation. Semir B* et al.* conducted a set of experiments on the basis of HFD-fed mice gaining considerable weight. As a result, HFD-derived intestinal stem cells (ISCs) showed enhanced capacity to initiate organoids in a niche-independent growth manner, and generated more secondary organoids with multilineage differentiation 24. Simulation of HFD, *ex vivo* treatment of mouse and human intestinal organoids with fatty acid constituents reproduced the results* in vivo*, indicating the excellent performance of organoids* in vitro* to recapitulate protocols. Mechanically, robust evocation of PPAR-δ program was observed in HFD-fed mice or fatty acids- exposed organoids. Enhanced PPAR-δ signaling in intestinal progenitors enforced not only organoid-initiating capacity but also tumor-initiating potential 24.

Another research probed into the interactions between adipocytes and colonic cancer cells in organoids. Tumor organoids were generated from intestinal tumor tissues isolated from APC/KRAS compound mutant mice and cultured in 3D Matrigel with modifications. During progression of organoids co-cultured with adipocytes, tumor cells proliferated and dedifferentiated. In-depth study revealed that the transfer of free fatty acids released from adipocytes to cancer cells promoted tumor organoids growth. Along with tumor cells dedifferentiation, the expression of WNT target genes (e.g., Lgr5 and Cd44) associated with 'stem-like' colon cancer cells were remarkably elevated in tumor organoids. In contrast, the expression of genes related to epithelial cell differentiation, such as sucrase-isomaltase (Sis) and mucin 2 (Muc2), was significantly decreased. These results demonstrated that adipocytes favoring the dedifferentiation and aggressiveness of colon cancer cells so as to drive tumorigenesis and promote tumor progression 25.

Additionally, obesity‐related systemic inflammation has been reported to act in colorectal tumor initiation. *In vivo* experiment, obese mice tended to have more colorectal premalignant lesions and colorectal tumors, and showed an enhanced IL‐13 concentration in blood. When IL‐13 was administrated to normal colon organoids, morphological capacity of organoids significantly increased in a time- and concentration‐ dependent manner. These findings indicated that inflammation intrigued by obesity, notably the augmented production of IL‐13, could play an important role in the carcinogenesis of obesity‐associated CRC 26.

#### Cholesterol consumption

Cholesterol consumption has been correlated with gastrointestinal cancer occurrence in epidemiological studies 44. Providing excess dietary cholesterol or driving endogenous cholesterol synthesis promotes ISCs proliferation *in vivo* 27. Although many previous studies have indicated that the neoplastic transformation might be triggered by phospholipid remodeling, the detailed mechanisms that phospholipases facilitate tumor initiation and growth remains unclear.

Capitalized on organoid technology, Bo Wang* et al.* revealed an intriguing link between membrane phospholipid remodeling and cholesterol biosynthesis, to make sure of the act of cholesterol as a mitogen for ISCs. Hyper-proliferation of ISCs has been detected in lysophosphatidylcholine acyltransferase 3(Lpcat3)-deficient mice. Isolation of the intestinal crypts of Lpcat3-deficient mice, Lpcat3-deficient organoids were established and embedded in Matrigel followed by cultured in a crypt culture medium. Significantly, Lpcat3 deletion increased the size, number, and complexity (higher number of buds) of organoids, with enriched genes related to cholesterol biosynthetic. Pharmacologically blocking cholesterol synthesis alleviated crypt hyperproliferation in Lpcat3-deficient organoids. In addition, disrupting Lpcat3-dependent phospholipid and cholesterol homeostasis dramatically enhanced tumor formation in Apc^min^ mice. These findings may shed a light on a critical dietary-responsive phospholipid-cholesterol axis regulating ISC proliferation and tumorigenesis 27.

#### Red meat consumption

The dietary uptake of heme iron mainly from red meat is a probable carcinogen to humans as it increases the risk of developing CRC 45. The underlying malignant transformation mechanisms include intestinal hyperproliferation 46, 47, gut microbiota alteration 48, 49 and genotoxic effects 50, 51. In a recent study, the optimized intestinal organoid was introduced to investigate the genotoxic and cytotoxic potential of heme iron in colonic epithelial cells. After the culturing of isolated murine intestinal crypt in Matrigel with complete culture medium, intestinal organoids were established followed by exposing to incremental levels of hemin or ferric chloride (0-100 μM) for 24 h. Hemin significantly reduced viability of normal human colonic epithelial cells and murine intestinal organoids, whereas CRC cell lines were modestly affected. Hemin triggered reactive oxygen species (ROS) generation and led to oxidative DNA damage mediated by Nrf2 signaling and heme oxygenase-1 (HO-1) stimulation preferentially in non-malignant intestinal epithelial cells rather than in CRC cells. Comparatively, inorganic iron displayed weak toxicity in all cell models as well as the intestinal organoids. Taken together, nature heme iron, instead of inorganic iron, led to genotoxicity and cytotoxicity of normal colonocytes, which might boost the outgrowth of neoplastic cells *in vivo* and fuel the intestinal carcinogenesis 29.

#### Vitamin E

Anti-oxidants are considered as chemo-preventive agents against tumorigenesis as they scavenge ROS to protect DNA from damage and to maintain genomic stability 52. However, the contrary findings of Selenium and Vitamin E Cancer Prevention Trial (SELECT) showed an unexpected increased risk of prostate cancer with Vitamin E supplementation 53. Another study indicated the protective effect of Vitamin E probably for advanced prostatic cancer (PCa) but not for latent or early PCa 54. To elucidate the paradox effect of Vitamin E on PCa evolution, researches were conducted based on benign (primary), premalignant and malignant prostate epithelial organoids. As the results highly reminiscent of the clinical data from SELECT, Vitamin E significantly enhanced the growth and survival of premalignant organoids, while the effect was counteracted with Selenium supplementation. Indeed, Vitamin E treatment endowed premalignant organoids with pro-tumoral gene expression signature that was suppressed by Selenium exposure or combination with Vitamin E. Conforming to the observations in clinic, SELECT supplements decreased cell proliferation and induced cell death in cancer organoids, yet exhibited no adverse impact on the propagation of benign organoids. The distinctive characteristic of premalignant organoids was that the detached cells showed low ATP levels due to diminished glucose uptake and glycolysis. In comparison, malignant cells continued to proliferate even if they detached. In the normal control, cells were nurtured depending on the intact tumor-derived extracellular matrix (ECM) attachment. Intriguingly, Vitamin E increased the survival of premalignant organoids through stimulating fatty acid oxidation, which was the other major pathway for energy generation during tumorigenesis 28.

### Alcohol consumption

Growing evidence consolidate a strong relationship between alcohol consumption and increased CRC risk 55. Matthew Devall *et al.* modeled a normal colon organoid under prolonged alcohol exposure. The normal colon organoids were derived from colon crypts isolated from colon biopsies through routine colonoscopy of healthy patients. Over the passages in growth media, organoids gently dissociated from larger ones to pellets, and propagated within fresh growth media plus ethanol (2 μl per 1 ml of growth media). Persistent ethanol exposure was ensured by replacement of mixed media every 1 for 3 days. Ethanol treatment dose and exposure time were determined to mimic the circulating blood alcohol levels in daily regular drinkers 30. This organoid-based pilot study elucidated the potential molecular mechanisms underlying the consistent ethanol exposure as a risk factor of CRC. Using next generation sequencing approaches, the significant differentially expressed genes (DEGs) and open chromatin peaks were detected in response to ethanol treatment, disclosing candidate genes relevant to ethanol-associated CRC initiation for further research 56. However, this study was limited ascribe to the inclusion of organoids derived from only right colon of three male subjects. Investigation was extended to the organoids came from either left or right normal colon biopsies of different individuals without gender bias. With the same ethanol exposure between organoids of different colon locations, significant DEGs were observed in left colon organoids versus the right organoids. Among these significant DEGs, genes that mark colonocytes and goblet cells were reduced. In contrast, marker genes for enteroendocrine cells, stem cells and transit amplifying (TA) cells were increased, and more pronounced in left colon organoids than the right colon organoids under ethanol treatment. This shift in cell population towards increased stem and TA cells at the expense of reduced differentiated cells created conditions optimal for cell division and probable mutations that would potentially develop into cancer 57. The results of alcohol as a risk factor for left CRC are consistent with the observation from epidemiological studies 58, 59.

The consequences of alcohol abuse are far beyond. As is well-known that chronic alcohol intake can disrupt intestinal barriers, which increases the risk of endotoxemia and gut-derived local or systemic inflammation. These conditions are implicated in multiple pathological diseases, of which almost half of global liver cirrhosis and malignancy in a subset 60-62. Studies of the mouse small intestine organoids exposing to ethanol have demonstrated that alcohol deterred stem cell proliferation and subsequent organoid growth by inhibiting the Wnt/β-catenin pathway 63. The alcohol-induced detrimental effect on intestinal barrier integrity is long-lasting and likely to increase the possibility of bacterial translocation. For future investigations, using organoid techniques such as microfluidics to co-culture gut and liver organoid along with milieu components, such as immune cell populations, may offer an organ-on-chips platform for evaluating gut-liver axis in liver cancer 64.

### Aging

The association between aging and cancer is complex. Recent studies have shown that DNA methylation-based measures of biological aging are associated with increased risk and shorter survival of multiple cancers including colorectal, gastric, kidney, lung, prostate and urothelial cancers 65. Previous studies reported that the increasing spontaneous promoter DNA hypermethylation-associated genes were silenced during senescence and in early tumorigenesis. Due to lack of suitable preclinical model, the role of aging in cancer development is still underexplored. Organoid, which accumulate epigenetic changes over the long-term culture, appears to mimic *in vivo* aging- like epigenetic alterations. It provides a permissive platform for oncogenic transformation to study the links of senescence with cancer predisposition.

BRAFV600E-mutant colon adenocarcinoma (COAD) is a representative scenario with extensive abnormal gene-promoter CpG-island (CGI) methylation or the methylator phenotype (CIMP). Yong T *et al.* employed the Braf^V600E^ proximal colon organoid to simulate COAD organoid formation, illustrating the precursor role of aging-like spontaneous promoter DNA hypermethylation in favoring tumorigenesis. The Braf^V600E^ proximal colon organoids were established from proximal intestinal crypts of mice carrying heterozygous Cre-activable Braf^V600E^ 66. After 11 weeks of culturing in medium with all niche factors (EGF, Noggin, R-Spondin 1, and Wnt3a, abbreviated ENSW), Braf^V600E^-activated (Braf^CA^) organoids exhibited profound morphological changes and neoplastic dysplastic features. In 5 months, all Braf^CA^ organoids replicated independent of niche factors, and performed progressive properties of stem cells and an accentuating polypoid growth phenotype (Braf^CA-IND^). Notably, the levels of methylation were distinctly higher in Braf^CA-IND^ organoids compared to Braf^CA^, which indicated that the methylation changed gradually instead of acutely after BrafV600E induction. Such associated genes that silenced led to WNT pathway activation, giving a stem-like state retainment and differentiation defects. As a result, aging organoids were more likely to undergo BrafV600E transformation than younger ones, eventually presenting the characteristics of human proximal BRAFV600E-driven colorectal adenocarcinoma with extensive, abnormal CGI DNA methylation, or CIMP 31. It revealed a link between aging-like epigenetic abnormalities and predisposition to oncogene-driven colon tumorigenesis. Organoid is considered as an ideal model for identification of epigenetic and genetic changes in the context of aging-related tumorigenesis, as well as for exploration of cancer prevention and interception strategies. For further studies, organoid models are expected to inquiry whether aging-like events in organoids truly reflect senescence condition in humans.

### Environmental chemicals

Organoid-based chemical carcinogenesis has been modeled recently. Expose of organoids derived from mouse normal lung/liver/mammary tissues to 4 genotoxic chemicals, including acrylamide (AA), ethyl methanesulfonate (EMS), diethylnitrosamine (DEN) and 7,12-dimethylbenz[a]anthracene (DMBA), no notable morphological changes were observed in organoids with each chemical additive. Followed by inoculation into dorsal subcutaneous of female nude mice, organoids presented tumorigenicity and progressed to lung, liver or mammary cancers. Under EMS or AA treatment, wildtype or Trp53-silenced lung organoids displayed typically carcinogenic characteristics such as activation of oncogenic kinases, as previously demonstrated in the nodules from the nude mouse subcutis. Vicious transformation was observed in liver (biliary tract) organoid under DEN exposure 32. Noteworthy, mammary tissue-derived organoids with DMBA treatment and Trp53 knockout exhibited tumorigenicity, in contrast to those of wild-type Trp53. Similar results were obtained in parallel *in vivo* experiments 67.

During malignant transformation, the genotoxic carcinogens may induce initial genetic events in the organoids, proceeding to intrigue tumorigenic potential. Therefore, treatment of normal tissue-derived organoids with chemicals are helpful of tracing the critical molecular events in the carcinogenesis of specific tumor types. Organoids featured with shorter periods and carcinogen-targeted organs/tissues may outperform those animal carcinogenesis models. Application of organoid carcinogenesis model could exclude the effects of the intraindividual environment such as chemicals metabolism and distribution. Besides, it involves the tissue microenvironment, including interactions between target parenchymal cells and nonparenchymal cells in tumor formation 68.

### Microorganism

Microorganisms have been implicated in approximately 20% of human malignancies 69. The correlation of some microbes with tumorigenesis has been evaluated in organoid system, such as bacterial infection like *Escherichia coli* (*E. coli*) and Enterotoxigenic Bacteroides fragilis (ETBF) for CRC, *Helicobacter pylori* (*H. pylori*) for gastric cancer (GC), and *Salmonella enterica* for gallbladder cancer (GBC) 70-72, Virus infection like Hepatitis B Virus (HBV) for hepatocellular carcinoma (HCC) and human papillomavirus (HPV) for cervical cancer.

#### Microbiomes in Intestinal cancer

*E. coli*, enriched in CRC biopsy than in healthy mucosa, has been suspected to directly promote invasive carcinoma in genetically susceptible hosts. The majority of *E. coli* isolated from CRC mucosa carry the pathogenicity genomic island pks (pks(+) *E. coli*), which encode a set of enzymes responsible for the synthesis of colibactin. Colibactin, a genotoxic compound for alkylating DNA on adenine residues and inducing double-strand breaks (DSBs) in cultured cells, potentiates intestinal tumorigenesis in mice and has recently been implicated in colorectal carcinogenesis. To deep understand the mutagenic characteristics of *E. coli*, pks(+) *E. coli* strain originated from a CRC biopsy was micro-injected into the lumen of intestinal organoid 73. An isogenic clbQ knockout strain (pks^∆clbQ^ *E. coli*) incapable of producing active colibactin was set as negative control. Just for one day, the pks(+) *E. coli* induced DNA damage as DSBs and inter-strand crosslinks (ICLs) in exposed epithelial cells. Over 5 months of pks(+) *E. coli* exposure achieved by repeated injections into single cell-derived organoids, genotoxic colibactin caused a distinct mutational signature with increased pks-specific single base substitution (SBS) levels and a bias towards T>N substitutions in epithelial cells. In addition, a featured small indel signature with single T deletions at T homopolymers was also induced by colibactin administration 33.

In recent studies, mutant versions of TP53 (mutations in R172H and R270H) were found to have contradictory roles in different segments of the gut, with oncogenic effect expected in the distal gut versus tumor suppressive effect in the proximal. Eliran K* et al.* have demonstrated these gain-of-function mutations in TP53 actually conferred the tumor-suppressive effects driven by disruption of the WNT pathway in mutant mice and organoids. Attaching importance to the environmental microorganisms that sparse in the proximal and dense at the distal gut, Lynch SV *et al.* found that the tumorigenic predisposition in the distal gut of p53 mutant mice was largely due to the fact that WNT inhibition mediated by p53 mutants could be reversed by abundant gut microbiota 74. Since no significant difference was detected in microbial flora between wild-type mice and p53^R172H^ mice, it is reasonable to speculate that bacteria-derived metabolites modulate susceptibility to cancer formation. By using organoid culture system, host-microbe interaction was monitored during tumorigenesis. The intestinal organoids were generated from mouse derived crypts of the jejunum or ileum with different genotypes. In corresponding organoid models, properties of Jejunal and ileal were kept well, with higher presentation of Lct levels in jejunal and and Fabp6 ileal organoids 75. The hyperactivation of WNT/β-catenin pathway in 4OHT-induced organoids was achieved by culturing in R-spondin-absent medium. Gallic acid, a metabolite of gut microbiota that reproduce the microbiota effect, significantly enhanced the proliferation capacity and override WNT-blocking status of p53 ^R172H^ organoids 34. It is assumed that other microbiota-derived metabolites or indirect metabolite effects might also modulate mutant p53 and its associated functions. Accordingly, dietary management and metabolites antagonists may serve as preventive and therapeutic options for cancer.

Accumulating human and animal experiment evidence suggests that EBFT plays a role in colitis and colon tumor formation through producing B. fragilis toxin (BFT) 76-78. Tumor formation is fueled by a pro-inflammatory signaling cascade, which undermining the epithelial barrier integrity at the beginning. While treating mice colon-derived organoids with BFT, an intestinal glucosylceramide sphingolipid was substantially elevated in response to BFT -induced colon epithelial cell signaling 79. As BTF is supposed to upregulate ROS levels that lead to DNA damage and mutations 80, interpreting the regulatory mechanism of EBFT in colon organoids may help elucidate EBFT associated colon tumorigenesis.

Conversing to numerous microbiomes escalating the CRC risk, it is attractive that some intestinal probiotics, such as Lactobacillus and Lachnospiraceae, are protective strategies for tumorigenesis. The latest research conducted by Naoki S et al. have shown that Lactobacillus gallinarum (L. gallinarum) inhibited CRC growth *in vitro* and intestinal tumorigenesis *in vivo*. Specifically, L. gallinarum culture-supernatant, identified as indole-3-lactic acid (ILA), significantly promoted apoptosis in CRC cells and patient-derived CRC organoids, but not in normal colon epithelial cells 81. Therefore, gut microbiota modulation has been considered as a promising tactic to prevent and treat CRC. Therapies like probiotics, antibiotics and fecal microbiota transplantation are alternative options for altering gut microflora arrangement 82.

#### *H. pylori* in Gastric cancer

Intensive studies have consolidated the strong link between *H. pylori* infection and gastric cancer occurrence over decades. *H. pylori*-infected organoid, established successfully by microinjecting *H. pylori* into pre-organized gastric organoids (gastroids), provides a more comprehensive mechanobiological platform to probe into the microbes-related gastric carcinogenesis. Based on such mouse derived *ex vivo* 3D system, Wroblewski *et al.* found that cytotoxin-associated gene A (CagA) stood out in oncogenic properties induced by* H. pylori*. More specifically, *H. pylori* promoted epithelial cells proliferation through reduction and mis-localization of claudin-7 at the tight junction, in a H. pylori virulence factor CagA and β-catenin-dependent manner, driving the infection-related carcinogenesis 35. McCracken* et al.* have broadened these findings by *de novo* generation of human gastroids. *H. pylori* infection resulted in a rapid association of CagA with the c-Met receptor, activation of signaling and induction of epithelial proliferation 36.

*H. pylori*-derived CagA contributed to an epithelial-to-mesenchymal transition (EMT)-like phenotype in epithelial cells (e.g., loss of cell polarity and adhesion, increased cell motility) 83, 84. By using human gastroids, Buti *et al*. found that the apoptosis-stimulating protein of p53 2 (ASPP2), a host tumor suppressor and an important CagA target, promoted the CagA-positive *H. pylori* bacteria survival. CagA-ASPP2 protein interaction facilitated remodeling of the partitioning-defective (PAR) polarity complex and led to cell polarity loss. Further research uncovered the activated RTK/PI3K/AKT signaling pathway in the CagA-ASPP2 interaction. Interfering this protein-protein interaction by inhibitors of RTK/ PI3K/AKT signaling or a CagA-binding ASPP2 peptide efficiently prevented the loss of polarity and dampened the survival of *H. pylori* in infected organoids 84.

The transition from gastritis to gastric cancer driven by *H. pylori* infection is relevant to increased levels of pro-inflammatory mediators, including reactive oxygen and nitrogen species that induce DNA damage 85-87. The production of H_2_O_2_, during the process of converting polyamine spermine to spermidine mediated by spermine oxidase (SMOX) 88, 89, creates DNA damage in gastric cells 90, 91. Previous studies have demonstrated that *H. pylori* infection enhances the expression of SMOX in human and rodent gastric tissues, which is associated with oxidative DNA injury 86, 92 and predisposition of gastric carcinogenesis. Johanna C. Sierra* et al.* generated Smox^-/-^ gastric organoids from Smox-deficient mice with conspicuous lower gastric spermidine levels. In such organoids, *H. pylori*-induced inflammation and carcinogenic signaling were attenuated, as well as DNA damage and β-catenin activation levels. Then the pathogenicity model of *H. pylori* infected human gastric organoids was established in the presence or absence of novel SMOX inhibitors 37. Parallelly, *H. pylori* infection elevated spermidine levels, while the effect was abrogated by the administration of the SMOX inhibitor. Based on the analysis in combination of human and mouse gastric-derived organoids, *H. pylori* infection increased spermidine levels in a SMOX-dependent manner, giving rise to β-catenin oncogenic signaling activation. Thus, targeted inhibition of SMOX could be considered as a chemo-preventive strategy for gastric cancer.

#### *Salmonella enterica* in Gallbladder cancer

Chronic carriage of *Salmonella enterica* serovars Typhi and Paratyphi A is clinically associated with GBC. However, the potential molecular mechanism of this fatal connection remains unclear. Mice model is insufficient as the murine serovar *Salmonella Typhimurium* lacks the human-produced typhoid toxin. Differently, gallbladder organoids (GBOs) are valuable for exploring the principles of the oncogenic transformation facilitated by *Salmonella Paratyphi A*. In a recent study, GBOs were generated from isolated primary epithelial cells of human and murine gallbladder and maintained in a defined Matrigel. Every 7 to 10 days followed by enzymatic and mechanical shearing, the GBOs were passaged and seeded for further expansion in fresh Matrigel. Of note, long-term maintenance of GBO cultures depended on the presence of R-spondin, an activator of the Wnt/β-catenin signaling pathway and a crucial factor for regeneration of the gallbladder epithelium. The reliable representation of the GBO was confirmed with the architecture and the main molecular features resembling the organ *in situ*. To duplicate the pathology of cancer-associated bacterium S. Paratyphi A infection, organoids were mechanically sheared to expose the luminal side and cocultured with *Salmonella enterica serovar Paratyphi A*. Through such procedures, Ludovico P. Sepe* et al.* observed that bacteria could invade epithelial cells and cause the host cell's DNA injury. Relying on typhoid toxin CdtB subunit, extensive DNA damage occurred and extended to the neighboring non-infected cells. By cultivating the organoid-derived cells into polarized monolayers in the ALI system, longer term infection model was available to investigate into the fate of chronically infected cells. Bacterial infection was apparent at the beginning and attenuated later, indispensable of typhoid toxin. Along with the anti-apoptotic effects of *Salmonella* on host cells and persistent inflammation, the accumulating DNA damages were likely to contribute to the incremental risk of developing malignant mutations in chronic carriers. These may partly explain that chronic carriers of typhoid *Salmonella* serovars have higher risk of gallbladder cancer 40.

#### HBV in HCC

HBV with stringent host species and cell-type specificity is a leading cause of chronic liver cirrhosis as well as HCC. Epidemiologic evidence identifies heterogeneous outcomes across the patients with HBV infection on the basis of individual various genetic background. Due to lack of suitable infection models, research on the pathogenesis of HBV was sluggish. In a recent study, human induced pluripotent stem cells (hiPSCs)-derived functional liver organoid was established by co-culturing hiPSC-derived endodermal, mesenchymal, and endothelial cells within a chemically defined medium in a 3D microwell culture system. With self-organized capability, these cells ultimately differentiated into a functional organoid that was naturally readily infected with HBV 41, 93. The novel organoid model allows for long-term HBV propagation, recapitulating the viral lifecycle and virus-induced hepatic dysfunction. Founded on this virus-host model, Nie* et al.* suggested that the HBV infection impaired hepatic gene expression, decreased ALB secretion, augmented the levels of ALT and LDH in infected hepatic cells. Moreover, HBV infection has been considered as a driving force for EMT during liver cancer evolution, with remarkably elevated expressions of EMT markers including Snail Family Transcriptional Repressor 2 (SNAI2) and Twist Family BHLH Transcription Factor 1 (TWIST1) in infected liver organoids. IFNα and IFNγ partially restored the innate immune activation *in vivo*, greatly suppressed virus replication but stimulated additional hepatic injury in hiPSC liver organoids 41. The achieved liver organoid culture affords a promising platform for studying the impact of heterogenous genetic background on virus-induced host outcomes and for developing personalized treatment for HBV and even HBV-induced hepatocarcinogenesis.

#### HPV in cervical cancer

Oncogenic HPV, such as HPV16 and HPV18, is a main risk factor of cervical cancer, including almost all squamous cell carcinoma (SCC) and approximately 85% of adenocarcinoma (ADC). Yet despite the prevalence of HPV in female, only few of them develop malignancy. It has been realized that persistent HPV infection escaping from host immune surveillance give rise to the neoplastic transformation, while most infected cells are eliminated by the immune system. To interrogate the crosstalk between viral and early cell-based innate immune system, the immune-competent model was established founded on *in vitro* organoid. Langerhans cells (LCs), act as the immune first line to strive for eliminating the carcinogenic HPV16, have been concerned 42. Epithelial organoids have been generated from patient-derived cervical biopsies or commercially primary cervical cells including keratinocytes and fibroblasts. The number of HPV-positive keratinocytes was controlled by the Cre-Lox recombination system 94, 95 and screened thereafter. It was intriguing that altering the proportion of infected cells within the epithelium actually modeled the varying stages of the disease, ranging from low-grade to high-grade lesions 96. Immersing of fibroblasts in a collagen matrix was defined as dermal equivalent to support the keratinocyte growth. Importantly, LCs were seeded on top of the dermal equivalent at a ratio of 1:1 compared to the number of keratinocytes. Although LCs accounting for approximately 2 - 3% of the epithelium normally, superfluous LCs were added as most of them would be lost due to the less residing efficacy in the 3D scenario. After maintained in Matrigel for several days, these organoids were transferred into an ALI created with the dermal equivalent condition. Using a modified transwell migration assay, the timely maturation of LCs could be traced through assessing LC characteristics and measuring HPV effect qualitatively. Such immune-competent organoid system is accurately tailored for the crucial time-window of early virus elimination in a complex organism and is prospect for elaborating the mechanisms of HPV variants influence on disease development 42.

So far, most HPV infection models typically utilize undifferentiated keratinocytes as host cells. The metaplastic epithelium of the transformation zone especially the squamocolumnar junction (SCJ) of the uterine cervix is the major target of HPV invasion with disposition towards cancer evolvement. To prioritize the cervical carcinogenic model caused by HPV, normal SCJ organoids have been established to give rise to carcinogenesis naturally. The organoids were derived from patient's normal SCJ region that were surgically co-resected with non-cervical gynecologic tumors. Both columnar and squamous epithelium within the region was macroscopically estimated. Using Matrigel bilayer organoid culture protocol, the organoids basically appeared to compact structure, dominantly made up of squamous cells. However, in a subset of organoids, a small population of mucin-producing endocervix cells co-existed with the squamous cell population in a back-to-back manner, which greatly resembles the configuration of the actual SCJ. This research defined a group of cuboidal SCJ cells existing in organoids, only as a minority of mucin-producing endocervix cells. The obtained organoids appeared to imitate the constitution and gene expression of cervical SCJ cells along with metaplastic squamous cells, providing a novel platform for HPV-driven cervical cancer development 97.

Herfs et al. reported the discovery of SCJ cells, a residual embryonic cell population located at the SCJ that are progenitors for reserve 98, 99. Furthermore, the specific gene expression profiles analysis predicted that HPV-related neoplastic cells, whether SCC or ADC cells, were descendants of SCJ cells, strongly supporting SCJ cells to be the cell origin of HPV-driven cervical carcinogenesis. As almost all available cervical cancer organoids have been designed to study the carcinogenesis of cervical SCC, Mengzhu Z et al. established an *in vitro* carcinogenesis organoid model of ADC through modifying molecular background of SCJ cells. Differentially expressed genes candidate for inducing ADC lineage differentiation were identified through TCGA data analysis. Therefore, the carcinogenesis model of ADC could be acquired by generation of organoid cultures with normal human cervical keratinocytes immortalized with ectopic TERT expression (HCK1T), which conditionally expressing the combination of HPV oncogenes (HPV16 E6E7 or HPV18 E6E7), genetic alterations commonly found in ADC and potential lineage-specifying factors (c-MYC, KRAS^T58A^, FOXA2, SMAD4mi). The results showed that exogenous FOXA2 acting as a lineage-determine factor can trigger differentiation or conversion of HCK1T toward the columnar cell lineage from the squamous cell lineage. In organoids expressing FOXA2 with SMAD4 reduction, the mucin- producing cells were increased compared to exogenous FOXA2 alone, implying that reduced SMAD4 may promote FOXA2-driven columnar cell line differentiation 100.

### Inflammatory bowel diseases

Inflammatory bowel diseases (IBDs), usually presented as ulcerative colitis (UC) or Crohn's disease (CD), are at higher risks of developing colitis-associated colorectal cancer. Particularly in UC patients, the risk of CRC occurrence raises up to 20-30 folds than general population 101. The advent of organoid culture gives insights into the role of long-term inflammation in the malignant transformation of intestinal epithelial cells (IECs). To mimic chronic inflammation* in vitro*, normal intestinal organoids derived from mice colon were propagated in the medium supplemented with cytokines and bacterial components mixture for over a year. Subsequent gene set enrichment analysis indicated the activation of NF-κB signaling pathway in treated organoids. Following long-term inflammatory stimulation with cytokine treatment for 11 weeks, NF-κB signaling activity was maintained and ROS was still detectable. Gene expression profile in the organoids at 60 weeks after reagents removal was similar to that at 12 weeks after reagents removal, suggesting irreversible cell transformation under the long-term inflammation 43.

## Prospect of the organoid model in tumorigenesis

**Figure 2:**
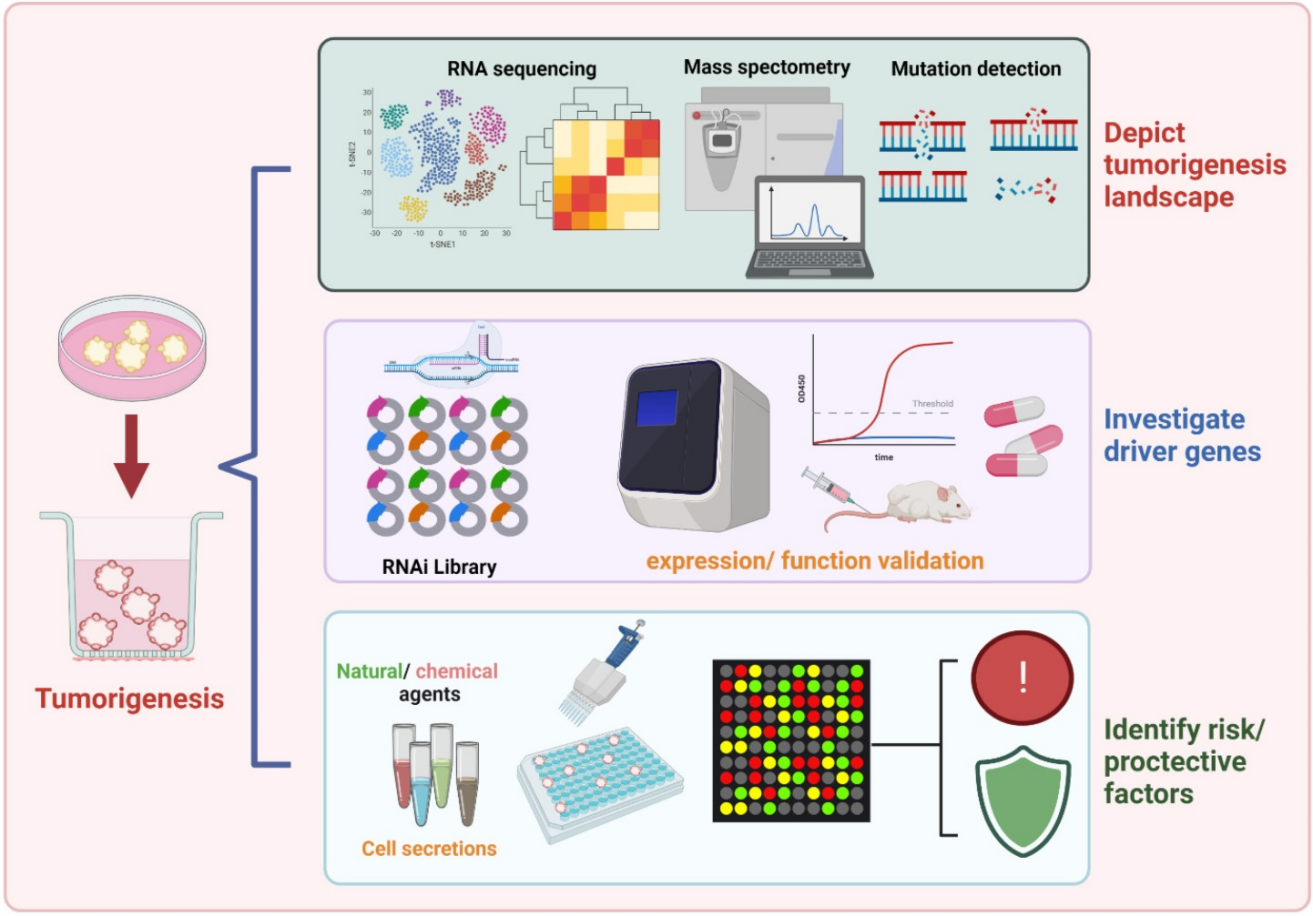
** The prospect of organoid model in favoring carcinogenesis exploration.** Existing evidence have focused on identifying cancer risks for specific tumor types. Relying on the optimizing organoid model, future studies may better understand the landscape of tumorigenesis, screen robust carcinogenesis-biomarkers, and development of novel preventive agents.

Oncogenesis is driven by genomic mutations of somatic cells and evolution of alterations in tissues microenvironment on the relative influence of inherent cell divisions and external exposures. Over the decades, epidemiological studies have focused on the determination of environmental and genetic factors that favor malignant tumor development and progression. Based on the organoid model, it is of great importance to depicting the landscape of tumorigenesis, identifying robust biomarkers, and developing novel preventive agents (**Figure 2**).

Organoids derived from normal wild-type tissues, with the congenital “clean” genetic background, provide an ideal platform for discovering initial events of tumorigenesis. Preservation of primary epithelium en bloc with TME components, organoid culture allows for better control of the cellular milieu, better spatial organization of cell types, and improved mimicry of *in vivo* cell behavior, which is suitable for modeling the tumorigenesis influenced by variables and probable pro-tumor factors. On lineage-tracing studies in CRC carcinogenesis with organoids conducted by Hiroki K et al., pericryptal leptin receptor (Lepr)-lineage cells proliferated to generate melanoma cell adhesion molecule (MCAM)+ CAFs that shape the tumor-promoting immune microenvironment. Preventing the expansion/differentiation of Lepr-lineage CAFs or inhibiting MCAM activity could be effective therapeutic approaches for CRC 102.

Cancer has the characteristic of histological and functional heterogeneity, arising from initial molecular events in the settings of dynamic micro-environment, resulting in a clinical challenge for patient treatment. Organoids represent a facile tool for studying natural tumor evolution with increasing heterogeneity, as they can be continuously manipulated and observed throughout the carcinogenesis process. Given the distinctive ability to differentiate to specific cell types, adult stem cell-derived organoids can be directed for enrichment of specific subtypes of cancer 33. There is a great promise that utility of organoid culture to capture the biological drivers of specific subtypes is favorable for personalized cancer treatment. For example, the molecular traits of serrated CRC were detected using a preclinical model of organoids. Two genes, Braf and Tgfbr2, lead to transmurally invasive adenocarcinoma. *De novo* tumorigenesis of patient-derived GC organoids was manipulated with CRISPR-Cas9 incorporation and expanded in a condition with specific cocktail of stem cell ''niche factors'' including WNT3A and R-spondin (WNT signal activators), EGF, FGF10, Noggin (a BMP inhibitor), and A83-01 (a TGF-b inhibitor). Ultimately, a range of GC organoids were generated that encompassed the histological, molecular, and phenotypic diversity of human GCs, including previously unestablished GS-GCs and hepatoid adenocarcinoma. Therefore, using patient-derived organoids combined with phenotype-based screening and CRISPR-Cas9 based reverse-genetics approach enables a comprehensive genome-wide study on subgroups of cancers to delineate the underlying molecular alterations.

## Conclusions

Carcinogenesis is a complex process involving a set of genetic alterations occurred sequentially in a given context. Under the influence of inherent tumor-susceptibility genes, cancer formation depends on endogenous cellular processes and is affected by exogenous factors such as diet, aging, environmental chemicals exposure, microbe infection, or inflammation. Herein, we highlight the employment of organoid culture system in determination of environmental factors either promoting or refraining tumorigenesis. Organoids are particularly available for better understanding of host-microbe interactions either increase or reduce the risk of cancer. Early intervention of tumorigenesis is thought to be an effective strategy for cancer prevention or favorable prognosis of cancer patients. In addition, specific subtypes of cancer can be modeled and identified by organoid culture to investigate the key molecular alterations important for each step in the carcinogenesis process. The novel organoid technology sheds light on the promising unique tumor drivers, biomarkers and specific druggable targets, bridging the gap between the genetics and the current biological clinical understandings.

## Figures and Tables

**Table 1:** Organoid models for studying risk factors in oncogenesis.

Risk factor	Cancer type	Organoid culture system					Parallel model	Reference
		Organoid derivation	Culture system	Risk exposure modeling	Phenotype alteration	Mechanism		
HFD	**CRC**	Mouse and human-derived normal ISCs	Matrigel	Supplement with fatty acid constituent (palmitic acid, oleic acid or a lipid mixture)	Enforced organoid-initiating capacity	Enforced PPAR-δ signaling	HFD-fed mouse model	24
		Intestinal tumor tissues of Apc /Kras compound mutant mouse	Matrigel	Co-culture with adipocyte	Dedifferentiation and aggressiveness of colon cancer cells	CPT1A mediated FAO	-	25
		Human normal colonic tissues	Matrigel	Supplement with IL-13 to mimic obesity associated inflammation	Morphological capacity changes in organoids	IL-13 mediated signaling pathway	Obesity Mouse model	26
Cholesterol consumption	**Gastrointestinal cancer**	Isolated intestinal crypts of Lpcat3-deficient mice	Matrigel	-	Increased size, number, and complexity of organoids	Dietary-responsive phospholipid-cholesterol axis	Apc^min^ Mouse model	27
Vitamin E	**PCa**	Benign, premalignant and malignant human prostate cells	Matrigel	Supplement with vitamin E	Enforced growth and survival of premalignant organoids	Activation of fatty acid oxidation	-	28
Red meat consumption	**CRC**	Isolated normal murine intestinal crypts	Matrigel	Supplement with hemin or ferric chloride	Reduced viability of the organoids	Enforced ROS generation and oxidative DNA damage mediated by Nrf2 signaling and HO-1 stimulation	Human colonic epithelial cells	29
Alcohol consumption	**CRC**	Colon crypts from human healthy biopsies tissue	Matrigel	Prolonged ethanol exposure	-	Induced chromatin accessibility and differential gene expression associated with malignancies	-	30
Aging	**BrafV600E- COAD**	Proximal intestinal crypts of BrafV600E-activated mice	Matrigel	Prolonged culture in medium with all niche factors	Stem-like state and a differentiation defects of accentuated polypoid growth phenotype	Abnormal CGI DNA methylation, or CIMP mediated by WNT pathway activation	-	31
Environmental risks	**Lung, liver and mammary cancers**	Lung, liver and mammary tissue of normal mouse	Matrigel	EMS, AA, DEN or SB treatment	No obvious changes *in vitro* and enlarged subcutaneous nodules in mouse	-	Mouse model	32
*E. coli*	**CRC**	Healthy human intestinal tissue	Matrigel	Microinjecting pks(+) *E. coli*/ pks^∆clbQ^ *E. coli*	DNA damage induced by pks(+) *E. coli*	Genotoxic colibactin induced SBS and ID signatures	-	33
Distal gut Microbiome	**p53^R172H^ CRC**	Crypts isolated from the jejunum or ileum of mouse	Matrigel	Gut microbiota-derived gallic acid	Enforced proliferation capacity of p53^R172H^ organoids	TCF4-chromatin interaction suppression and H3K4me3 modification at genomic WNT promoters	Mouse model	34
*H. pylori*	**GC**	Mouse stomachs tissue	Matrigel	Microinjecting *H. pylori*	CagA-dependent epithelial cell proliferation and mis-localization of occludin at the tight junction	CagA-dependent β-catenin nuclear translocation	Mouse model	35
		hiPSC-derived from antral tissue	Matrigel	Microinjecting *H. pylori*	CagA-dependent epithelial cell proliferation	Phosphorylation of c-Met receptor	-	36
		WT and Smox-/- mice	Matrigel	Supplement with *H. pylori*	Induced inflammation, DNA damage	Activation of β-catenin signaling mediated by SMOX	-	37
		Human gastric epithelial stem cells from surgical samples of human gastric corpus	Matrigel	Microinjecting *H. pylori*	Cell polarity loss	RTK/PI3K/AKT signaling pathway and partitioning-defective polarity mediated by CagA-ASPP2 interaction	-	38
		Human gastric epithelial stem cells from surgical samples of human gastric corpus	Matrigel	Microinjecting *H. pylori*	Gastric gland lineages mounted a strong inflammatory response in contrast to pit lineages	Activation of NF-κB pathway	-	39
*Salmonella Paratyphi A*	**GBC**	Primary epithelial cells of human and murine gallbladder	Matrigel, ALI	Co-cultured with *Salmonella enterica serovar Paratyphi A* or with its isogenic cdtB knockout strain	Typhoid toxin dependence host cell DNA damage	Typhoid toxin induced DNA double-strand breaks and genomic instability	-	40
HBV	**HCC**	hiPSCs	Matrigel	Supplement with HBV	Decreased ALB secretion, increased ALT and LDH levels, induced EMT phenotype	-	-	41
HPV	**Cervical cancer**	Human primary keratinocytes, co-cultured with fibroblast, LCs	Matrigel, ALI	Supplemented with HPV	Host immune evasion by HPV	-	-	42
Inflammation	**CRC**	IECs isolated from mouse colon	Matrigel	Supplement with a mixture of cytokines and bacterial components	-	Activation of NF-κB signaling pathway	-	43

**Abbreviation**: **HFD**: high fat diet; **CRC**: colorectal cancer; **ISCs**: intestinal stem cells; **CPT1A**: carnitine palmitoyltransferase I; **FAO**: fatty acid oxidation; Lpcat3: lysophosphatidylcholine acyltransferase 3; **PCa**: prostatic cancer; **ROS**: reactive oxygen species; **HO-1:** heme oxygenase-1; **COAD**: colon adenocarcinoma; **CGI**: CpG-island; **CIMP**: CpG island methylator phenotype; **EMS**: ethyl methanesulfonate; **AA**: acrylamide; **DEN**: diethylnitrosamine; **SB**: sodium benzoate; ***E. coli***: *Escherichia coli*; **SBS**: single base substitution; **ID**: small indel; **TCF4**: T cell factor 4; ***H. pylori***: *Helicobacter pylori*; **GC**: gastric cancer; **CagA**: cytotoxin-associated gene A; **hiPSC**: human induced pluripotent stem cells; **SMOX**: spermine oxidase; **ASPP2**: apoptosis-stimulating protein of p53 2; **HBV**: Hepatitis B Virus; **HCC**: hepatocellular carcinoma; **EMT**: tumor microenvironment; **GBC**: gallbladder cancer; **ALI**: air-liquid interface; **HPV**: human papillomavirus; **LCs**: Langerhans cells; **IECs**: intestinal epithelial cells.

## Abbreviations

TME: tumor microenvironment
PDOs: patient-derived organoids
ECM: tumor-derived extracellular matrix
EGF: epidermal growth factor
HFD: high fat diet
CRC: colorectal cancer
ISCs: intestinal stem cells
ROS: reactive oxygen species
COAD: colon adenocarcinoma
CIMP: CpG island methylator phenotype
CGI: CpG-island
SELECT: Selenium and Vitamin E Cancer Prevention Trial
PCa: prostatic cancer
AA: acrylamide
EMS: ethyl methanesulfonate
DEN: diethylnitrosamine
*E. coli*: *Escherichia coli*
ETBF: Enterotoxigenic Bacteroides fragilis
H pylori: *Helicobacter pylori*
GC: gastric cancer
GBC: gallbladder cancer
DSBs: double-strand breaks
BFT: B. fragilis toxin
EMT: epithelial-to-mesenchymal transition
ASPP2: apoptosis-stimulating protein of p53 2
SMOX: spermine oxidase
HBV: Hepatitis B Virus
HCC: hepatocellular carcinoma
hiPSCs: human induced pluripotent stem cells
GBO: gallbladder organoids
HPV: human papillomavirus
LCs: Langerhans cells
SCJ: squamocolumnar junction
IBDs: inflammatory bowel diseases
UC: ulcerative colitis
IECs: intestinal epithelial cells
Lepr: pericryptal leptin receptor
MCAM: melanoma cell adhesion molecule
